# Some Great Hospitals of America

**Published:** 1917-04-28

**Authors:** 


### SOME GREAT HOSPITALS OF AMERICA.

### BRITISH TYPES AND NEW DEPARTURES.

It is not generally understood in this country that many of the older, and amongst them some of the best administered, hospitals of the United States were originally founded, and are still maintained, by funds given for some or all the following purposes: endowment, site, buildings, equipment, maintenance. That is to say, there are a considerable number of hospitals in the United States which rely largely on voluntary gifts, or are maintained by funds presented by wealthy Americans and devoted to hospital purposes.

The hospital idea has made great progress with the people throughout the United States during the last thirty, and especially in the last twenty, years. One of the most important influences for good in the hospital sense was the planning and opening of the Johns Hopkins Hospital at Baltimore, which is under the government of twelve trustees elected for life—a self-perpetuating body. The late Dr. John S. Billings was successful with his scheme and plans for this hospital, which were selected by the trustees after a limited competition. Dr. Billings, during his life, was the great hospital authority of the United States, and as his life, which we reviewed in The Hospital of June 19, 1915, p. 251, demonstrates, he was a man of fine character, fulness of knowledge, and rare ability. He was fortunate in having the co-operation of Dr. Henry M. Hurd as superintendent of the Johns Hopkins Hospital—a man of the quietest presence and most far-reaching influence which it has ever been our pleasure to meet.

It is not necessary to describe in detail the wonderful completeness of the Johns Hopkins Hospital, closely associated as it is with the Johns Hopkins University. This hospital is unique in the circumstances of its original planning and creation, its splendid efficiency from its opening to the present time, and the object-lessons it affords to every visitor in proportion to the knowledge and experience of hospitals, and the treatment of disease, which each may possess. We gave a fairly complete account of its position, principal features, and excellence on page 339 *et seq.* of The Hospital for January 27, 1917. We will only repeat that we have often felt, and never more forcibly or certainly than during the visit of inspection we paid to this hospital on November 1, 1916, that no hospital administrator's or practitioner's education is complete, until he has mastered the Johns Hopkins Hospital system and methods, and fully absorbed their atmosphere. As a pioneer of progress it has continuously fulfilled the objects which inspired its inception, which have been faithfully and exhaustively pursued and developed during the twenty-seven years of its existence. The Superintendent, Dr. Winford H. Smith, is full of knowledge, his grasp of every detail is remarkable, and an inspection under his guidance yields continuous profit and pleasure.

Most of the greater cities have interesting types of large hospitals, many of them affiliated to universities, or medical schools, or both, and the nursing organisations and training-schools associated with them have done yeoman service in raising the standard of nursing and encouraging its development. They have secured a continuous growth in the quality of their nurses' education and class training, which constitute such predominant features in the best types of hospitals throughout the United States.

Philadelphia has some thirty hospitals, of which the Pennysylvania Hospital, founded in 1751, contains most features of interest—an interest which is increased by the publication of "The History of the Pennsylvania Hospital, 1751 to 1895," by Thomas G. Morton, M.D., senior surgeon and president of the medical staff. This history contains many reproductions of ancient charters and documents of importance and value, as well as some excellent engravings, and constitutes altogether a book worthy of preservation in the libraries of antiquarians, as well as of hospital men who take a wide interest in the history and development of the field of work to which they may devote their lives. Some of the old wards in the Pennsylvania Hospital have been cleverly converted or reconstructed, leaving many of the old features without interfering in any way with their hygienic and general efficiency for the accommodation and treatment of patients. The town hospital, an older establishment, contains 2,000 beds, with an average number occupied of 1,852; its expenditure is about £140,000 a year. In addition to several special hospitals there is also the hospital of the University of Pennsylvania, founded as recently as 1874, of which an Englishwoman, Miss Marion E. Smith, is the superintendent. It contains upwards of 400 beds, and has at present much accommodation for paying patients of the better class, which seems to be popular and is usually, we understand, fully occupied.

New York is a great city for hospitals of all kinds, sizes, and qualities. The majority of them have been founded within the period—nearly fifty years—which has elapsed since the writer first became superintendent of a hospital. This fact is interesting as showing that the growth, improvement, and multiplication of hospitals dates from about the same period in the great cities of the United States and this country. The New York

Polyclinic Hospital, with 205 beds, which is worked on the combined free and pay system, possesses features of interest. Its medical staff, for instance, consists of eighty-eight visiting physicians and surgeons, and forty-three specialists, in addition to resident surgeons. There is a good opening in New York for the provision of a modern, up-to-date hospital of the first rank, planned with infinite care and knowledge by the best authorities America contains, including Dr. S. S. Goldwater, superintendent of the Mount Sinai Hospital. Dr. Goldwater has spent a most useful and busy life; his knowledge of hospital construction and planning is exceptionally good, he has taken infinite pains to perfect his knowledge and mastery of hospital construction, administration, and everything calculated to benefit the citizens of New York hygienically, and to minister to their health, comfort, and wholesome, uplifting, and pleasurable existence. Dr. Goldwater's career, which is only now entering on what will probably prove to be its most interesting stage, is one worthy of study, because it is full of instruction for those who can appreciate good and thorough work, pursued with a single eye to securing the best and most efficient results, through municipal and institutional reform, under the control of men who have no axes to grind and are whole-heartedly desirous of securing the best possible results, at a reasonable cost, with the avoidance of waste and the absence of " graft " of all kinds.

Boston is a city full of interest, too, to all institutional and social workers of every type. England has learnt much and wisely from Boston institutions, and Boston has reciprocated the compliment, like many other American cities, by studying British and Continental methods and assimilating their best features when making developments for their own uplifting. The Massachusetts General Hospital, founded in 1811, decades of years before the majority of other hospitals were thought of, still maintains its supremacy and interest. Boston owes much, and many things, to the Massachusetts General Hospital, and under its skilled, active, and knowledgeable administrator Dr. Frederick A. Washburn, it is extending and improving the character of its work, which cannot fail to prove of supreme interest to all hospital people the world over. The Massachusetts General Hospital owes much of its prosperity to the generosity of the citizens of Boston. Its close association with Harvard University did much to cement and develop the efficiency and reputation of its medical school.

In recent years the Peter Bent Brigham Hospital has been established. It was founded by P. B. Brigham, who left the residue of his property in 1877 to accumulate, for twenty-five years from his death, in the hands of trustees, at the end of that period to be used in the founding of a hospital for the care of sick persons in indigent circumstances residing in the County of Suffolk. This led to prolonged discussion in the courts, which ended in March 1911. Dr. J. S. Billings was engaged to give expert advice; a committee of experts was appointed, of whom he was one, six architects were invited to compete and present plans, and contracts for the construction of a hospital with accommodation for 200 patients were signed in August 1911, and completed in July 1913. Dr. H. B. Howard, formerly superintendent of the Massachusetts General Hospital, had been appointed superintendent in April 1908, when he visited Europe, and the hospital buildings were erected largely under his supervision and control. We published an illustrated account of this hospital in our issue of December 30, 1916, pp. 261-263. It is closely associated with Harvard University, and the widespread popularity and great experience of Dr. Howard have brought it prominently into notice, while special points in its construction have led to interesting discussion and are till under trial. Both the Massachusetts General and the Peter Bent Brigham hospitals owe their existence to the generosity of citizens of Boston, and are examples of a type of American hospital which we defined at the commencement of this article.

Boston contains another hospital of a distinct type, founded in 1864. It is called the Boston City Hospital, and is financed and managed by trustees who, in compliance with the ordinances, report fully each year to the Mayor of the City of Boston, who is the channel of communication between themselves and the City Council, from which the hospital receives substantial aid every year. The City Hospital has attained, under able superintendence, a high state of efficiency, and is one which British visitors should not fail to inspect and study during a visit to the United States.

Did space permit, we should like to describe several other important American hospitals, including some of the more recently built hospitals in various States, notably that of Cincinnati, Ohio, and many other types, including the City and County Hospital at St. Paul, Minn. (700 beds), which has the unique distinction of being a municipal institution with a department for paying patients, and the Lakeside Hospital, Cleveland, Ohio (275 beds), founded in 1866 and rebuilt in 1896-98, of which Dr. A. R. Warner is the able acting superintendent. This latter institution will well repay a visit. It contains several good features, and its superintendent is a keen administrator, knowledgeable and courteous.

Most of the great cities have one or more well-administered hospitals, some of which in recent years have attained a position which makes them interesting to hospital workers. We unfortunately have only space to take a very few examples of the larger hospitals representing the three types of endowed, public-supported, and municipal institutions. These must suffice for our present purpose, which is to demonstrate how closely certain British and American types are identical in origin, and the financial foundations on which they rest. Administratively, the best British and American hospital has often many things in common, the great difference in administration being that in the United States every important hospital and many of the smaller ones—which latter are full of interest and often excellently managed—are placed under the control of a superintendent as the supreme head, the majority of whom are members of the medical profession. There are, too, a few laymen; and in hospitals with as many as 300 beds there are instances where the superintendent is a woman whose management frequently is highly efficient and well worthy of study. We look forward to a time, after the war, when intercommunication between English-speaking people all over the world will become so greatly facilitated, as to make it a common practice for leaders of thought, and the most competent and knowledgeable authorities in every department of professional, institutional, educational, and scientific life, to visit their brothers and sisters across the seas, to interchange views and experiences, and in this way to immeasurably hasten the full development, on the highest standard, of human progress amongst English-speaking people all the world over.

